# The Effect of FSH-Induced Nuclear Exclusion of FOXO3/4 on Granulosa Cell Proliferation and Apoptosis of Hen Ovarian Follicles

**DOI:** 10.3390/genes16050500

**Published:** 2025-04-27

**Authors:** Jinghua Zhao, Yuhan Sun, Simushi Liswaniso, Hengsong Wu, Xue Sun, Chunchi Yan, Ning Qin, Rifu Xu

**Affiliations:** 1 College of Animal Science and Technology, Jilin Agricultural University, Changchun 130118, China; zhaojinghua@ccsfu.edu.cn (J.Z.); sunyh990406@163.com (Y.S.); smliswaniso@gmail.com (S.L.); wx15298288966@163.com (H.W.); xuesun1128@163.com (X.S.); 18302478816@163.com (C.Y.); qinninglove2008@126.com (N.Q.); 2 School of Life Sciences, Changchun Normal University, Changchun 130032, China

**Keywords:** chicken, FOXO3/4, nuclear exclusion, cell proliferation, granulosa cell

## Abstract

Background: Follicle stimulating hormone (FSH) is key regulator for follicular development, differentiation, and maturation, and the effects involve various intra follicular factors, such as members of the forkhead box O (FOXO) subfamily. However, the specific role and mechanism of FOXO3 and FOXO4 in growth and development of hen follicles by affecting granulosa cell (GC) division and FSH response function are still unclear. Method: This study selected GCs from 6–8 mm chicken follicles, and immunofluorescence and Western blot methods were used to detect FSH-induced FOXO3/4 phosphorylation and nuclear exclusion. Quantitative real-time PCR and flow cytometry were used to investigate the regulatory effects of FSH-induced FOXO3/4 phosphorylation and nuclear exclusion on follicular GC proliferation, differentiation, and apoptosis. Results: This study found that the level of p-FOXO3/4 protein significantly increased in cells treated with FSH for 12 h, while the expression level of non-phosphorylated FOXO3/4 significantly decreased. After co-treatment with 10 ng/mL Leptomycin B (LMB), FOXO3/4 phosphorylation was effectively prevented. The immunofluorescence results showed that FOXO3 and FOXO4 were originally distributed in the GC nucleus and cytoplasm, whereas they were almost accumulated in cytoplasm when treated with FSH for 12 h. Conversely, FOXO3/4 nuclear translocation was blocked by LMB. Moreover, RT-qPCR and flow cytometry results showed that FSH treatment significantly increased proliferation and differentiation of cells but significantly reduced GCs apoptosis. However, LMB also eliminated these stimulating or inhibitory effects on cell proliferation. Conclusion: These findings provide new evidence that FSH-induced FOXO3/4 nuclear exclusion promotes GCs proliferation and reduces GCs apoptosis during hen follicular development.

## 1. Introduction

The development of ovarian follicles plays a vital role in poultry reproduction, influencing both clutch characteristics and egg production [1]. This process largely relies on the proliferation, differentiation, and apoptosis of granulosa cells (GCs) and theca cells in addition to the growth and maturation of oocytes within the hen’s ovary [2,3,4]. A variety of gonadotropins and their receptors, for instance, follicle-stimulating hormone (FSH) and FSH receptor (FSHR), are also implicated in GC differentiation, maturation and steroidogenesis, and follicle development and selection [5,6,7]. In granulosa cells, FSH serves as the primary hormonal trigger for the maturation of ovarian follicles and is also a crucial regulator of steroid hormone production [8,9]. Its biological functions are activated through the interaction with its specific FSHR receptor in poultry [10]. FSH has been demonstrated to encourage the selection of follicles that exhibit the highest levels of FSHR mRNA expression within the granulosa layer of specific pre-hierarchical follicles ranging from 6 to 8 mm in diameter [5]. Additionally, it influences the proliferation and differentiation of granulosa cells, which are responsible for synthesizing progesterone and estrogen, which are essential for the growth and maturation of preovulatory follicles. This process is facilitated by the up-regulation of StAR and CYP11A1 and steroid 17-α-hydroxylase (CYP17) expression in the ovaries of hens [3,11]. The function and regulatory mechanism of FSH in ovarian follicle growth, differentiation, and maturation through its interaction with FSHR to activate a number of downstream effectors and intracellular signals has been well documented [2,12,13,14,15], but characterization of FSHR expression in the GCs of ovarian follicles induced by FSH remain to be further clarified. Furthermore, a wide variety of transcription factors, for example, the members of forkhead box O (FOXO) subfamily, have been proven to be part of ovarian follicle development as well as ovarian function through response to FSH signals [16,17,18], and the FOXO members mainly function to promote GC apoptosis and follicular atresia [16,17,19]. However, FSH plays the opposite role by facilitating GC proliferation and follicle growth, selection, and maturation [3,11,20,21]. Therefore, the contribution of the FOXO members to FSH signaling cascades has been attracting more and more researcher attention.

The members of the FOXO family regulate many cellular programs, including cell proliferation, apoptosis, cell cycle transitions, autophagy, and responses to oxidative stress, which play a significant role in deciding cell fate [22,23,24]. In mammalian ovaries, FOXO3 were found to regulate follicular atresia by promoting GCs apoptosis [16,25]. FOXO3 is a pro-apoptotic molecule: while FOXO3 protein was found to be rich in granulosa cells of early atretic follicles during atresia, the expression levels of FOXO3 mRNA in GCs of porcine ovaries rose [16]. Moreover, in patients with polycystic ovary syndrome (PCOS), researchers found a strong positive correlation between GC apoptosis and total FoxO3 levels; however, a negative relationship was observed with the phosphorylated FOXO3 protein (p-FOXO3) levels [19]. Furthermore, the recent study revealed that FOXO3 was expressed in the follicles throughout all phases, and with rising follicle hierarchy, the expression level in follicles climbed, which promotes GC apoptosis of hen ovarian follicles [18]. Similar to other members of FOXOs, FOXO4 has been proven to contribute substantially to controlling cellular proliferation, cell cycle arrest, and apoptosis in mammals [26,27,28]. And FOXO4 functions in the mechanism of cellular nuclear translocation and transcriptional regulation, along with its isoform FOXO3 factor, although their molecular component and structure are divergent from each other [29,30]. FOXO4 can be phosphorylated by PI3K/PDK1 signaling, causing its inactivation and nuclear exclusion [31,32]. Without PI3K/PDK1 activation, FOXO4 can be found in the nucleus, where it serves as a transcription factor [33]. Recently, it was revealed that the expression of FOXO4 is in the strict control of microRNAs, and the subcellular localization of FOXO4 depends on phosphorylation modification [26]. Thus, the nuclear re-localizations and functions of FOXO4 are firmly commanded by different signaling pathways that depend on the various physiological states or cellular processes.

However, there is a scarcity of studies examining how follicle-stimulating hormone (FSH) regulates the FOXO3 and FOXO4 factors and its subsequent effects on the granulosa cells of chicken follicles. This experiment examined the role of FSH in regulating the phosphorylation and nuclear exclusion of the FOXO3 and FOXO4 proteins in granulosa cells of chicken follicles via the FSHR. Furthermore, it investigated how the exclusion of FOXO3 and FOXO4 from the nucleus affects the proliferation, differentiation, and apoptosis of these granulosa cells. In this study, we provide evidence to explain and support the claim that FSH induces phosphorylation and nuclear exclusion of the FOXO3/4 in granulosa cells of hen ovarian follicles. The FSH-induced nuclear exclusion of FOXO3/4 leads to the inhibition of cell apoptosis but increases proliferation and differentiation of granulosa cells. Based on current understanding, for the first time, this study presents novel data on the nuclear exclusion of FOXOs by involvement of FSH in hen ovarian granulosa cells. The current finding has established a solid basis for further understanding the molecular regulatory mechanism of FOXO3/4 nuclear exclusion in the follicular development and atresia of the chicken ovary.

## 2. Materials and Methods

### 2.1. Ethics Statement

In this study, all protocols carried out on chickens received approval from the Institutional Animal Care and Use Committee (IACUC) at Jilin Agricultural University in Changchun, China. The animal experiments adhered to the ARRIVE guidelines [34]. Prior to organ removal, the chickens were euthanized in accordance with the IACUC guidelines for experimental subjects (Permission No. GR (J) 19-030). The process of euthanizing the hens adhered completely to the relevant Chinese laws and regulations regarding laboratory animal care and use, which are based on the State Council of the People’s Republic of China’s Regulations for the Administration Affairs Concerning Experimental Animals (2017 Revision). We implemented all possible measures to reduce the animals’ discomfort.

### 2.2. Sampling and Granulosa Cell Culture

A commercial variety of Lohmann Brown hens for laying was kept in laying batteries following the standard husbandry methods outlined in earlier reports [35]. A total of 22 hens were obtained from the population and euthanized when they reached 21 weeks of age; subsequently, follicles measuring 6–8 mm in diameter were promptly harvested. GCs from follicles were then isolated and cultured following a protocol that we reported earlier [4]. A cell suspension was then prepared from the isolated GCs before adding M199 culture medium (Biosharp Life Sciences, Anhui, Hefei, China) containing 10% of newborn calf serum, 100 IU/mL penicillin, 75 IU/mL streptomycin, and 10 ng/mL insulin. The GCs were then incubated in 5% CO_2_ and 95% air at 37 °C. Cells were treated with 10 ng/mL FSH (R&D Systems, Shanghai, China) for 0 h, 8 h, 12 h, and 24 h, and then, expression levels of the FSHR gene were quantified. To evaluate expression of the genes (such as FOXO3/4, etc.) directly induced by FSH, cells were maintained in culture with or without 10 ng/mL FSH for a duration of 12 h, either in the presence or absence of 10 ng/mL Leptomycin B (LMB; Beyotime, Shanghai, China).

### 2.3. Immunofluorescence Staining

In the process of cell culture, six-well tissue culture plates were filled with circular glass coverslips, which were placed there to promote cell growth. Following this, 4% paraformaldehyde (PFA, Beyotime, Shanghai, China) was used to fix the cells, and an immunostaining barrier pen (Biosharp Life Sciences, Anhui, Hefei, China) was applied uniformly along the edges of the coverslips to establish a 1 mm oil barrier. The cells were subsequently incubated with primary antibodies, namely anti-FOXO3 (Proteintech, Wuhan, China, diluted 1:500) and anti-FOXO4 (Bioss, Beijing, China, diluted 1:500), at a temperature of 4 °C for a duration of 12 h. Following the washing of cells with PBS, the samples were incubated with a goat anti-rabbit IgG antibody labeled with a fluorescent marker (Epizyme Bio-tech, Shanghai, China), diluted 1:1000. The visualization of the nuclei was accomplished with 4′,6-diamidino-2-phenylindole (DAPI, Epizyme Biotech, Shanghai, China). A laser confocal microscope (Zeiss) was used to capture all immunofluorescence images.

### 2.4. Western Blot Analysis

The procedure made use of a RIPA lysis buffer (Biosharp Life Sciences, Anhui, Hefei, China) to treat the cells for purposes of protein extraction from the samples. This was then proceeded by the adding of protease inhibitors cocktail (Epizyme Biotech, Shanghai, China) and phosphatase inhibitor cocktail (Epizyme Biotech, Shanghai, China), with subsequent sonication for fragmentation. The concentration of the protein was then adjusted following the instructions provided in the BCA protein assay kit (Beyotime, Shanghai, China). Nitrocellulose membrane transfer was then completed after electrophoresis at 150 V in a 10% SDS PAGE gel (Epizyme Biotech, Shanghai, China). Following the blocking step with 5% nonfat milk, the membranes underwent treatment with primary antibodies, which included anti-FOXO3 (Proteintech, Wuhan, China; diluted 1:2000), p-FOXO3 (Bioss, Beijing, China; diluted 1:1000), anti-FOXO4 (Bioss, Beijing, China; diluted 1:1000), p-FOXO4 (Bioss, Beijing, China; diluted 1:1000), and anti-β-actin (Boster Biological Technology, Wuhan, China; diluted 1:10,000) at 4 °C overnight. Following washing with PBS, the samples were then incubated with a goat anti-mouse IgG antibody linked to horseradish peroxidase for 1.5 h as the secondary antibody. Subsequently, images were captured and analyzed with an ECL Plus Western blotting detection system, in accordance with the manufacturer’s instructions.

### 2.5. Quantitative Real-Time RT-PCR

To assess the expression levels of mRNA for the target genes in the GCs, we conducted real-time quantitative reverse transcriptase PCR (qRT-PCR) as outlined in our earlier publication [35]. The materials utilized in this study are presented in Table 1. The reaction system included 10 μL SYBR, 0.4 μL upper primer, 0.4 μL lower primer, and 2 μL cDNA. The amplification protocol consisted of an initial step at 95 °C for 10 min, followed by 15 s at 95 °C, and an annealing phase at 60 °C lasting 60 s, with a total of 40 cycles. The 18S rRNA served as a normalizing gene.

### 2.6. Flow Cytometric Analysis

The cells processed as explained above were digested with trypsin without EDTA and the cells collected. After centrifugation, the supernatant was ridden, and the cells were washed with PBS, suspended again, and re-centrifuged to eradicate the supernatant. The level of apoptosis in granulosa cells from various treatment groups was detected using Annexin V/PI double staining. The prepared cell samples were stained according to the instructions of the annexin V-FITC/PI apoptosis test kit, which was instantly analyzed using the CytoFlex flow cytometer. In accordance with the scatter plot of the bivariate flow cystoscope, the proliferating live cells were displayed as (FITC−/PI−) in the lower left quadrant. Necrotic cells, also referred to as non-viable cells, appeared in the upper right quadrant and marked as (FITC+/PI+). It is thought by some that these cells represent non-viable apoptotic cells or what may be termed as dead cells, indicated by (FITC−/PI+). Apoptotic cells are shown in the right lower quadrant (FITC+/PI−).

### 2.7. Statistical Analysis

The SPSS 28 software package (IBM Corp, Armonk, NY, USA) was used for statistical analysis. Each experiment was performed a minimum of three times with samples collected from various batches of birds. To verify the normality of the data and ensure the relevance of parametric analysis, the Shapiro–Wilk test was utilized. Furthermore, Levene’s test was conducted to assess the homogeneity of variance among treatments during the execution of multiple comparisons. Since the analysis was based on samples from batches rather than the entire population, the interpretation of the *p*-value is crucial. When more than two groups were involved, a one-way ANOVA followed by Tukey’s multiple-comparison test was applied. A Student’s *t*-test was utilized after verifying that the distributions were normal, specifically when only treatment and control groups were compared. In the case of the values in treatment groups were compared to the control group, if the comparison was solely between each treatment group and the control group without comparing among treatment groups, the Dunnett’s test could have been an alternative approach. *p* < 0.01 or *p* < 0.05 was considered to be statistically significant.

## 3. Results

### 3.1. The Expression of FSHR mRNA in the GCs Regulated by FSH

**Figure 1:**
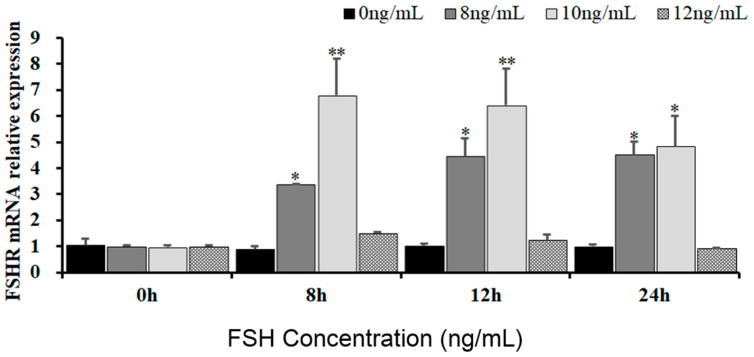
Expression of FSHR mRNA in undifferentiated granulosa cells is influenced by FSH. The GCs deriving from hen ovarian pre-hierarchical follicles measuring 6–8 mm in diameter were cultured at different concentrations of 0 ng/mL, 8 ng/mL, 10 ng/mL, and 12 ng/mL for durations of 0 h, 8 h, 12 h, and 24 h, respectively. Each treatment was replicated five times (*n* = 5), and the results are expressed as means ± SEM derived from these repeated experiments. Comparisons between the treatment groups and the control group were conducted using Dunnett’s test. The bars marked with superscript symbols indicate significant differences when compared to the control groups at ** *p* < 0.01 and * *p* < 0.05. Extremely significant difference: *p* < 0.01 **; significant difference: *p* < 0.05 *.

To reveal the effects of FSH signaling on the expression and phosphorylation of FOXO3/4 in the undifferentiated GCs by its interaction with the FSHR in the hen ovaries, the expression levels of *FSHR* mRNA in the GCs induced by FSH were firstly determined in the pre-hierarchical follicles of 6–8 mm diameter. As shown in Figure 1, expression levels of *FSHR* mRNA were dramatically elevated in the GCs cultured with 10 ng/mL FSH for 8 h or 12 h (*p* < 0.01). However, the levels of *FSHR* mRNA were then markedly declined in the GCs treated with 10 ng/mL FSH for 24 h (*p* < 0.05), although the *FSHR* mRNA expression levels were significantly higher compared with the controls (*p* < 0.05). Unexpectedly, the *FSHR* mRNA expression levels presented unchanged in the GCs under treatment with 12 ng/mL FSH from 0 h to 12 h compared with the control group (*p* > 0.05). These results provide evidence that FSH signaling activates its FSHR pathway, which may be involved in the regulation and function of FOXO family members in chicken ovarian follicle development.

### 3.2. FoxO3/4 Phosphorylation and Nuclear Exclusion Induced by FSH

**Figure 2:**
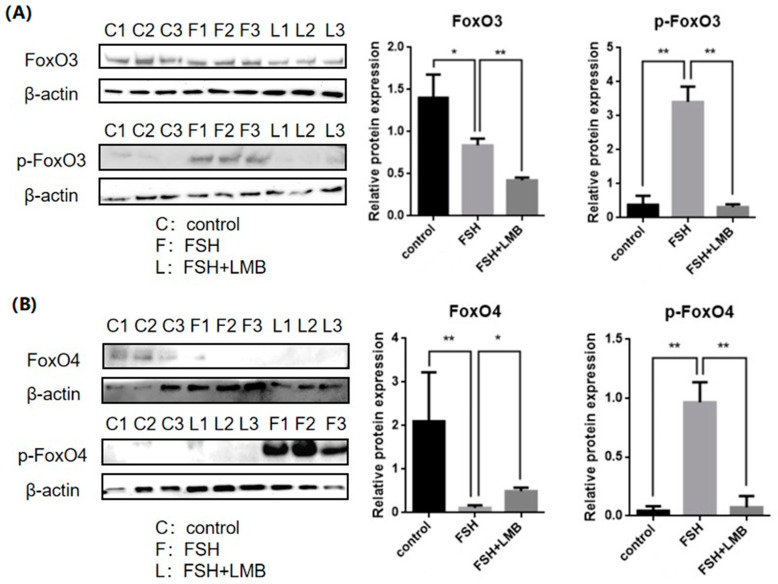
Effects of FSH on the FoxO3/4 phosphorylation in the cultured GCs of the ovarian follicles. (**A**) Levels of p-FOXO3 were evaluated through Western blot analysis in GCs that were treated with or without 10 ng/mL FSH for 12 h, in the presence or absence of LMB. (**B**) The p-FOXO4 levels were tested in the GCs under the treatment with or without the 10 ng/mL FSH for 12 h in the presence or absence of LMB. The data represent the mean ± SEM, (*n* = 5). Dunnett’s test, ** *p*  < 0.01 and * *p*  < 0.05. Extremely significant difference: *p* < 0.01 **; significant difference: *p* < 0.05 *.

**Figure 3:**
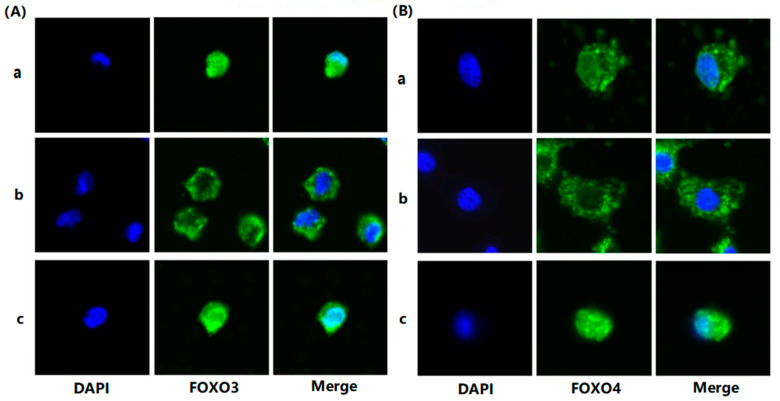
FSH-induced the nuclear exclusion of FOXO3/4 in the cultured GCs of the ovarian follicles. (**A**) Immunofluorescence detection of FOXO3 subcellular localization in the GCs. The GCs were treated with or without the 10 ng/mL FSH for 12 h in the presence or absence of LMB, which were stained using goat anti-rabbit FOXO3 (green). The nucleus was stained with DAPI (blue); scale bar = 5 μm. (**B**) Immunofluorescence detection of FOXO4 subcellular localization in the GCs. FOXO4 (green) and nucleus stained with DAPI (blue). a. control, without treatment of FSH or LMB; b. treatment of the cells with 10 ng/mL FSH for 12 h; c. co-treatment of the cells with 10 ng/mL FSH plus LMB for 12 h. *n* = 3 independent experiments. Leica DMI8, 600×; scale bar = 2.5 μm.

To evaluate the effects of FSH on the phosphorylation and nuclear exclusion of FOXO3/4 in granulosa cells derived from ovarian pre-hierarchical follicles, we examined the levels of phosphorylated FOXO3/4 (p-FOXO3/4) using the Western blotting technique. Additionally, the subcellular localizations of FOXO3/4 in cultured granulosa cells were assessed through immunofluorescence analysis. The results demonstrated that expression levels of FOXO3/4 protein were decreased significantly in the cultured GCs with the treatment of 10 ng/mL FSH and co-treatment of 10 ng/mL FSH and Leptomycin B (LMB) for 12 h compared to the control group (*p* < 0.01; Figure 2A,B). However, the levels of the p-FOXO3/4 were dramatically increased in the GCs under the FSH treatment for 12 h, and this stimulatory action of FSH was effectively prevented by the co-treatment with 10 ng/mL LMB (*p* < 0.01). Moreover, either between FOXO3 and FOXO4 proteins or p-FOXO3 and p-FOXO4, molecules exhibited expression patterns in the GCs greatly similar to FSH treatment, respectively. Furthermore, the double immunofluorescence staining revealed that total FOXO3/4 (including FOXO3/4 and p-FOXO3/4) was localized in the GC nucleus and cytoplasm, whereas the nuclear FOXO3/4 almost failed to be visualized in the nuclei but mainly accumulated in the cytoplasm of the GCs treated with FSH for 12 h. This indicates that the nuclear FOXO3/4 was re-localized to the cytoplasm of the cells upon induction of FSH signaling. Conversely, this FSH-induced nuclear translocation of the FOXO3/4 in the cultured GCs was greatly blocked by the LMB (*p* < 0.01; Figure 3A,B). These findings reveal that FSH promotes phosphorylation and concomitant nuclear exclusion of FOXO3/4 in the cultured GCs, which may directly bring about changes in the effects of FOXO3/4 on ovarian follicle development.

### 3.3. Expression of the Genes Associated with Cell Proliferation, Differentiation, and Apoptosis of the GCs Induced by FSH

**Figure 4:**
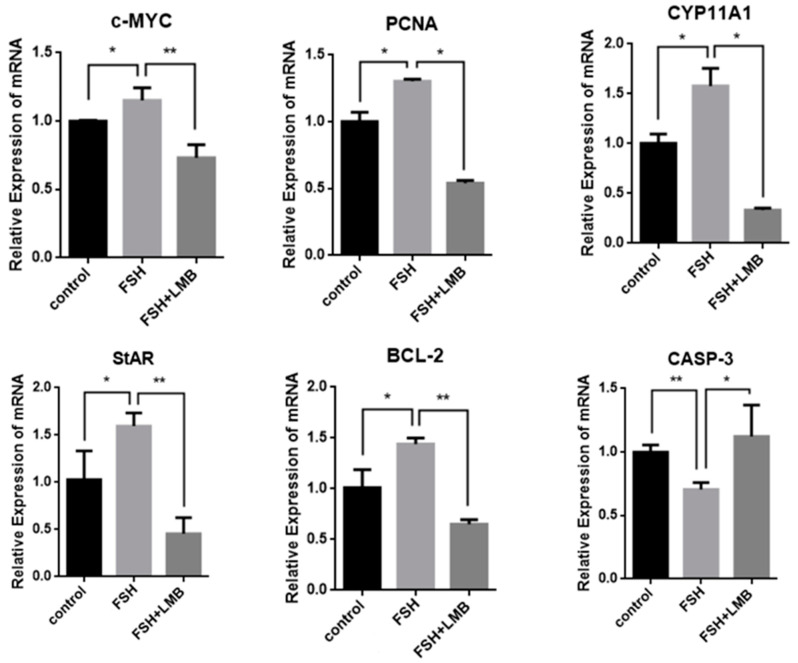
FSH-induced expression of *MYC*, *PCNA*, *StAR*, *CYP11A1*, *BCL-2*, and *CASP-3* genes in the cultured GCs. The data are presented as the means ± SEM from five replicate experiments, and bars with superscript symbol present the significant difference compared with control groups at ** *p* < 0.01 and * *p* < 0.05. Extremely significant difference: *p* < 0.01 **; significant difference: *p* < 0.05 *.

To further explore biological functions of the FSH-induced phosphorylation and nuclear exclusion of FoxO3/4 in ovarian follicle development and growth, the expression of a battery of candidate genes, including *MYC*, *PCNA*, *STAR*, *CYP11A1*, *BCL2*, and *CASP3*, that are tightly associated with cell proliferation, differentiation, and apoptosis of the GCs were examined. It has now been proven that the mRNA expression levels of the *MYC*, *PCNA*, and *BCL2* genes that are involved in follicle development by promoting cell proliferation and anti-apoptosis and the *STAR* and *CYP11A1* genes that serve as indicators for GC differentiation were dramatically enhanced in the GCs by the treatment of FSH, while the expression level of the *CASP3* mRNA was significantly reduced. However, all of these stimulatory or inhibitory effects induced by FSH on expression of the genes were predominantly abolished by the LMB (*p* < 0.01; Figure 4). These results indicate that FOXO3/4 are critical factors in promoting cell apoptosis and oppositely play roles in invigorating GCs proliferation and differentiation but reduce apoptosis in response to the FSH-induced phosphorylation and nuclear exclusion during ovarian follicle growth and development.

### 3.4. Effects of FSH-Induced Phosphorylation and Nuclear Exclusion of FoxO3/4 on GCs Proliferation and Apoptosis

**Figure 5:**
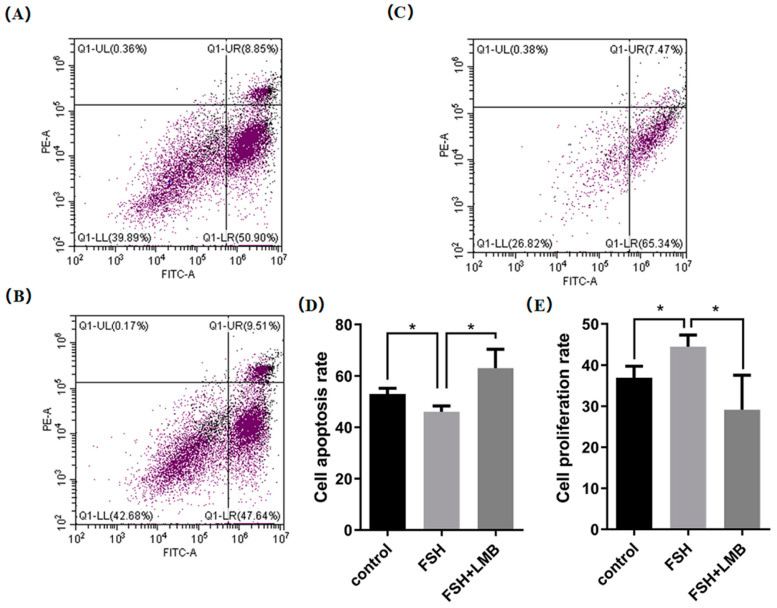
FSH-induced nuclear exclusion of FoxO3/4 on proliferation and cell apoptosis of the cultured GCs. The data are presented as the means ± SEM from three replicate experiments, and bars with superscript symbol present the significant difference compared with control groups at * *p* < 0.05. Significant difference: *p* < 0.05 *. Figures (**A**–**C**) show the flow cytometric analysis graphs of the 3 replicate experiments, figure (**D**) shows the cell apoptosis analysis bar graph and figure (**E**) shows the cell proliferation analysis bar graph.

Based upon the molecular roles of the FSH-induced phosphorylation and nuclear exclusion of FoxO3/4 in regulation of the aforementioned genes, flow cytometric analysis was used to scrutinize the proliferation and apoptotic rate of the cultured GCs with or without FSH treatment. As shown in Figure 5, a significantly higher cell proliferation rate and lower apoptotic rate of the GCs were observed in the FSH treatment group compared to the control group (*p* < 0.05). Nevertheless, these FSH-induced effects on the proliferation and apoptosis of the GCs were significantly abrogated by the LMB (*p* < 0.05). These results solidify the claim that the transcriptional factors FOXO3/4 function to promote GC proliferation and apoptosis via the FSH signaling-induced phosphorylation and nuclear exclusion of FOXO3/4 in the ovarian follicle growth and development of hens.

## 4. Discussion

The performance of chickens in laying eggs primarily depends on the progressive phases of ovarian follicle growth and maturation, where follicles measuring 6–8 mm are particularly significant [36]. FOXO3/4, as a transcription factor, affects the physiological processes of follicular granulosa cells through the regulation of downstream signaling factors in the nucleus [37]. The phosphorylation and nuclear exclusion of FOXO3/4 are regulated by the body through FSH, influencing the process. Nevertheless, there is a lack of relevant research on chicken follicular granulosa cells. This experiment researched how, in chicken follicular granulosa cells, FSH affects phosphorylation and nuclear exclusion of FOXO3/4. Based on the analysis of comprehensive experimental results, the addition of FSH promoted the phosphorylation of FOXO3/4 in chicken follicular granulosa cells, while phosphorylated FOXO3/4 remained in the cytoplasm and lost transcriptional activity. This process promoted the proliferation and differentiation of chicken follicular granulosa cells while inhibiting their apoptosis. The experimental results have molecular biological significance for studying egg-laying performance and follicle selection of chickens.

The WB results of this experiment demonstrate that the addition of FSH significantly increases the phosphorylation level of FOXO3/4 protein. After adding nuclear transport inhibitors, nuclear cytoplasmic transport of FOXO3/4 was blocked, resulting in its retention in cytoplasm and subsequent degradation by proteasomes [38,39]. At present, in the research on cell cycle inhibition of tumors and cancer cells, it has been found that FSH induced phosphorylation of FOXO3/4 and played a role in this process [40,41]. Inhibition of FOXO3 phosphorylation could lead to the transport of FOXO3 protein to the cell nucleus, activation of FOXO3 transcriptional activity, and eventually inhibition of the proliferation of liver cancer cells [42]. FOXO4 protein was found to be prevented from translocating into the nucleus after modifying, thus failing to activate the transcription of downstream target genes [33]. The comprehensive results of WB and IF indicate that the expression level of FOXO3/4 in the nucleus significantly dropped after action of FSH on chicken follicular granulosa cells, suggesting that FOXO3/4 undergoes nuclear exclusion upon phosphorylation. Studies have shown that FSH induces the direct phosphorylation of FOXOs in follicular granulosa cells through related signaling pathways [43]. Phosphorylation of FOXO3/4 proteins reduced their DNA binding activity and transferred them from the nucleus to cytoplasm by binding to 14-3-3 proteins [38,44]. AKT-mediated phosphorylation inactivated FOXO3/4 and promoted its localization in the cytoplasm [45,46]. Currently, relevant studies have focused on inhibiting nuclear exclusion of FOXOs and enhancing their proapoptotic effect as transcription factors, thereby inhibiting proliferation of cancer cells and tumor cells.

The process of FSH promoting FOXO3/4 nuclear exclusion has a promoting effect on the proliferation and differentiation of chicken follicular granulosa cells while inhibiting their apoptosis. FOXO3/4, as a transcription factor, has a pro-apoptotic effect [47,48,49]. FOXO3 could activate the apoptotic signaling pathway, inducing cell cycle arrest in the nucleus of human ovarian cancer cells, while FOXO3 in the cytoplasm is unable to serve as a transcription factor [50]. FOXO4 could promote cell apoptosis by down-regulating the anti-apoptotic gene BCL-2 [51]. Inhibition of FOXO4 up-regulation significantly increased the expression level of its downstream signaling molecule p27kip1, leading to G1 phase cell cycle inhibition and thus preventing cell proliferation [52]. After FSH treatment, FOXO3/4 protein was phosphorylated and retained in the cytoplasm, losing its nuclear transcription factor function, leading to down-regulation of cell cycle regulators, and inducing inhibition of cell proliferation and apoptosis [53,54]. Based on the results of RT-qPCR and flow cytometry, the findings of this study are consistent with those of earlier studies.

This study offers new evidence of the regulatory effect of FSH on FOXO3/4 and its effects on cell proliferation, differentiation, and apoptosis in follicular granulosa cells. However, there is still no research on the signaling pathway through which FSH acts upon FOXO3/4 in chicken follicular granulosa cells. Consequently, further experiments will continue to investigate the regulatory effects of related signaling pathways on FOXO3/4 protein.

## 5. Conclusions

In summary, the current study initially demonstrated that expression levels of the phosphorylated FOXO3 and FOXO4 were significantly increased in the cultured GCs from the ovarian follicles challenged by FSH in chickens, whereas the expression levels of non-phosphorylated FOXO3/4 markedly decreased. Simultaneously, FSH-induced nuclear translocation of FOXO3/4 in GCs was found, suggesting that phosphorylation of FOXO3/4 leads to their nuclear exclusion. Furthermore, the nuclear exclusion of FOXO3 and FOXO4 dramatically enhanced the expression of the *MYC*, *PCNA*, *BCL2*, *STAR*, and *CYP11A1* transcript levels but significantly reduced the expression of the *CASP3* mRNA level in a similar manner, which concomitantly resulted in a memorable augment in cell proliferation but a remarkable decrement in apoptosis of the GCs. These findings provide novel evidence that the FSH-induced nuclear exclusion of FOXO3/4 promotes GC proliferation while attenuating the apoptosis of GCs in hen ovarian follicles.

## Figures and Tables

**Table 1:** Primer pairs designed for quantitative real-time PCR analysis.

Gene	Forward Primer (5′-3′)	Reverse Primer (5′-3′)	Accession No.	Size
*FSHR*	ATGTCTCCGGCAAAGCAAGA	AACGACTTCGTTGCACAAGC	NM_205079.1	147 bp
*CASP-3*	ATTGAAGCAGACAGTGGACCAGATG	TGCGTTCCTCCAGGAGTAGTAGC	NM_204725.2	111 bp
*PCNA*	CTGAGGCGTGCTGGG	ATGGCGATGTTGCGG	NM_204170.3	133 bp
*StAR*	AGCAGATGGGCGACTGGAAC	GGGAGCACCGAACACTCACAA	NM_204686.2	147 bp
*CYP11A1*	CCGCTTTGCCTTGGAGTCTGTG	ATGAGGGTGACGGCGTCGATGAA	NM_001001756.1	111 bp
*c-MYC*	GAGAACGACAAGAGGCGAAC	CGCCTCAACTGCTCTTTCTC	NM_001030952.2	211 bp
*BCL-2*	CGCTACCAGAGGGAC	GAAGAAGGCGACGAT	NM_205339.3	135 bp
*PDK1*	AGACATCCCGAGCTACACCT	CGCCTTGGAAGTATTGTGCG	NM_001031352.4	81 bp
*SGK3*	TGCGTCCAGGAATCAGTCTCAC	AAGTCTGCTTTGCCGATCTTTCTC	NM_001030940.2	74 bp
*18s rRNA*	TAGTTGGTGGAGCGATTTGTCT	CGGACATCTAAGGGCATCACA	AF173612.1	169 bp

## Data Availability

The original contributions presented in this study are included in the article. Further inquiries can be directed to the corresponding author.
